# Search for Resistant Genotypes to *Cuscuta campestris* Infection in Two Legume Species, *Vicia sativa* and *Vicia ervilia*

**DOI:** 10.3390/plants10040738

**Published:** 2021-04-09

**Authors:** Eva María Córdoba, Mónica Fernández-Aparicio, Clara Isabel González-Verdejo, Carmela López-Grau, María del Valle Muñoz-Muñoz, Salvador Nadal

**Affiliations:** 1 IFAPA Centro Alameda del Obispo, Área de Genómica y Biotecnología, Apdo. 3092, 14080 Córdoba, Spain; clarai.gonzalez@juntadeandalucia.es (C.I.G.-V.); salvador.nadal@juntadeandalucia.es (S.N.); 2 Institute for Sustainable Agriculture-CSIC, Avda. Menendez Pidal s.n., 14004 Córdoba, Spain; z82logrc@uco.es; 3 Campus de Rabanales, University of Córdoba, E-14071 Córdoba, Spain; z82mumum@uco.es

**Keywords:** phytogenetic resources, parasitic weeds, field dodder, common vetch, bitter vetch, breeding for parasitic weed resistance, post-attachment resistance, sustainable crop protection

## Abstract

The dodders (*Cuscuta* spp.) are parasitic plants that feed on the stems of their host plants. *Cuscuta campestris* is one of the most damaging parasitic plants for the worldwide agricultural production of broad-leaved crops. Its control is limited or non-existent, therefore resistance breeding is the best alternative both economically and environmentally. Common vetch (*Vicia sativa)* and bitter vetch (*Vicia ervilia)* are highly susceptible to *C. campestris*, but no resistant genotypes have been identified. Thus, the aim of this study was to identify in *V. sativa* and *V.* *ervilia* germplasm collections genotypes resistant to *C. campestris* infection for use in combating this parasitic plant. Three greenhouse screening were conducted to: (1) identify resistant responses in a collection of 154 accessions of bitter vetch and a collection of 135 accessions of common vetch genotypes against infection of *C. campestris*; (2) confirm the resistant response identified in common vetch accessions; and (3) characterize the effect of *C. campestris* infection on biomass of *V. sativa* resistant and susceptible accessions. Most common vetch and bitter vetch genotypes tested were susceptible to *C. campestris.* However, the *V. sativa* genotype Vs.1 exhibited high resistance. The resistant phenotype was characterized by a delay in the development of *C. campestris* posthaustorial growth and a darkening resembling a hypersensitive-like response at the penetration site. The resistant mechanism was effective in limiting the growth of *C. campestris* as the ratio of parasite/host shoot dry biomass was more significantly reduced than the rest of the accessions. To the best or our knowledge, this is the first identification of *Cuscuta* resistance in *V. sativa* genotypes.

## 1. Introduction

The common vetch (*Vicia sativa* L.) is an annual legume native to the Mediterranean Basin. It is widely cultivated in many areas worldwide due to its high nutritional value as a grain legume or forage crop [1,2,3] and its ability to grow over a wide range of climatic and soil conditions [4]. Worldwide cultivation of common vetch area reached nearly 540,762 ha in 2018 with 34% of this area being cultivated in the Mediterranean Basin [5]. Spain is the main producing country of common vetch for which the growing area was 103,100 and 143,200 hectares for grain and forage production, respectively, in 2018 [6]. In traditional rain-fed areas in the Mediterranean Basin, common vetch is cultivated either as a monocrop or intercropped with cereals for improved forage harvesting and yield [7,8]. Bitter vetch (*Vicia ervilia* (L.) Willd.) is one of the oldest cultivated grain legume crops, originating in the Mediterranean and Middle East area [9,10,11]. Spanish cultivation of bitter vetch reached 54,900 ha during 2018 [12]. It is an annual, predominantly self-pollinated species, tolerant to marginal soils, and drought and cold climate conditions [13]. The inclusion of *V. sativa* and *V. ervilia* in crop rotations contributes to the increase in sustainability in agricultural systems, reducing the need for fertilizers and pesticides by improving soil fertility and reducing the incidence of pests and weeds [11,14]. The multiple benefits of *V. sativa* and *V. ervilia* are threatened by their high susceptibility to infection by parasitic weeds [15,16,17,18]. Despite the many advantages of the cultivation of these *Vicia* species in low input cropping systems, its cultivation is in decline, mainly due to the lack of investment in breeding programs to register elite cultivars [19].

The dodders (*Cuscuta* spp.) are stem parasitic plants from the Convolvulaceae family with none or reduced photosynthetic activity and as a consequence, they are obliged to obtain nutrients by parasitizing the stem of other plants [20]. The genus *Cuscuta* contains over 170 species distributed throughout the world [21]. Among them, *C. campestris* is one of the most damaging species worldwide for the agricultural production of dicotyledonous crops [22]. After germination, *Cuscuta* seedlings coil around host stems and differentiate prehaustoria that penetrate the host and connect to its vascular system for nutrient diversion [23]. The control of *Cuscuta* is difficult because of their persistent seedbanks formed by long living seeds with hard coats as well as their capacity to infect a broad host range including other weeds and the intimacy of haustorial connections with the host, which makes the application of control methods selective enough to kill the parasite without affecting the crop difficult [24,25,26]. For most crop-*Cuscuta* species pairs, control is limited or non-existent and for those crops where *Cuscuta* control is possible, the control strategies are mainly based in either phloem-mobile herbicides applied to herbicide-resistant crops [27,28,29] or infection-resistant crops [22]. Legume resistance to *Cuscuta* has only been reported in chickpea and the resistance mechanism is characterized by the failure of the prehaustorium to penetrate the host stem [22]. Resistance in *V. sativa* and *V. ervilia* has not been reported against the infection of stem parasitic weeds, but it has been frequently reported against the infection of root parasitic plants [15,16,17,18,30,31,32,33]. Resistant phenotype in vetch to *Phelipanche aegyptiaca* has been described with the appearance of a dark substance between host and parasite cells, and is associated with an increase in peroxidase activity and the increase in the concentrations of phenolics and lignin [30,31]. Resistant *V. sativa* to *O. crenata* is associated with lignification of the endodermal cells [32] and mucilage production inside vetch vessels, leading to the obstruction of the parasite nutritive supply [15]. Resistant *V. ervilia* to *Orobanche crenata* is associated with reduced induction of *O. crenata* germination and failure to the *O. crenata* haustorium to penetrate the host root [17,18].

*V. sativa* and *V. ervilia* are highly susceptible to *C. campestris*, but to the best of our knowledge, no resistant genotypes have been identified thus far in these crop species. Thus, the objective of this research was to identify resistant genotypes against *C. campestris* in two *Vicia* germplasm collections: a collection of 135 genotypes of *V. sativa* and a collection of 154 accessions of *V. ervilia*.

## 2. Results 

### 2.1. Search for Resistant Genotypes in Vicia ervilia

**Figure 1:**
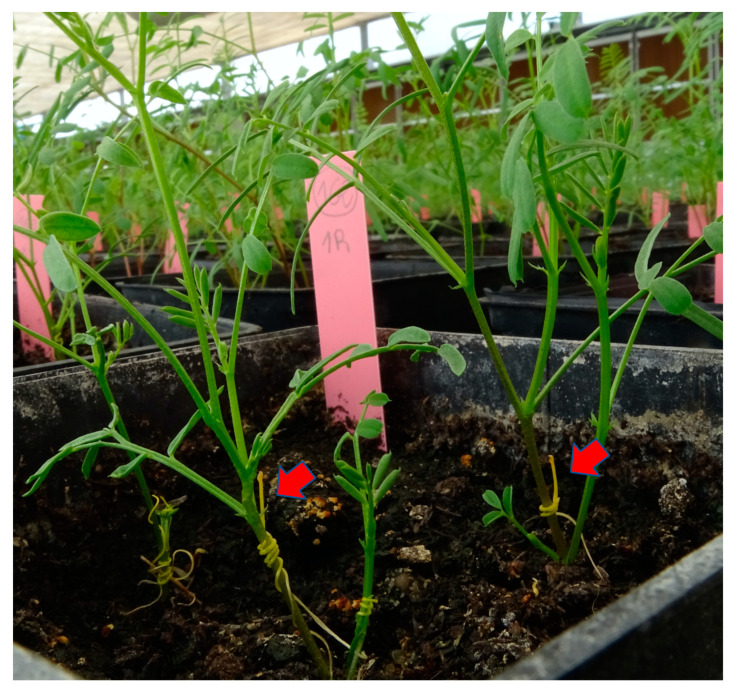
Susceptible response of *Vicia ervilia* plants. Red arrows point at the posthaustorial growth of nine day-old *Cuscuta campestris* seedlings

In order to identify *V. ervilia* genotypes with the capacity to inhibit *C. campestris* infection through resistance against haustorium formation and penetration, a collection of 154 *V. ervilia* accessions was studied in a greenhouse. Each *V. ervilia* accession was planted in triplicated pots and grown in a greenhouse for sixteen days before two day-old *C. campestris* seedings were placed around each *V. ervilia* plant. Table 1 shows that *C. campestris* seedlings were able to coil around the stem of all plants tested from each of the 154 *V. ervilia* accessions, revealing the absence of allelopathic mechanisms that could cause *C. campestris* repellency against the *V. ervilia* plants. Once the *C. campestris* stem is coiled around the host stem, prehaustoria forms and penetrates the stem to form vascular connections. From the nutrients extracted from the host through the vascular connections, *C. campestris* develops filamentous stems at the *Cuscuta-*host connection sites, called in this work posthaustorial growth (Figure 1). Table 1 shows the data recorded at seven days after inoculation in the 24 *V. ervilia* most resistant accessions in which at least 50% of the plants resisted the penetration of the haustorium and inhibited the posthaustorial growth of nine day-old *C. campestris* seedlings, with only one accession, the accession Ve.136, able to completely resist in all their plants the formation and penetration of the haustorium. The remaining 130 *V. ervilia* accessions allowed in more than 50% of their plants the development of nine day-old *C. campestris* posthaustorial growth. These differing responses among *V. ervilia* accessions observed against nine day-old *C. campestris* were not maintained in the successive days displaying all the *V. ervilia* accessions susceptible responses 14 days later.

### 2.2. Search for Resistant Genotypes in Vicia sativa

**Figure 2:**
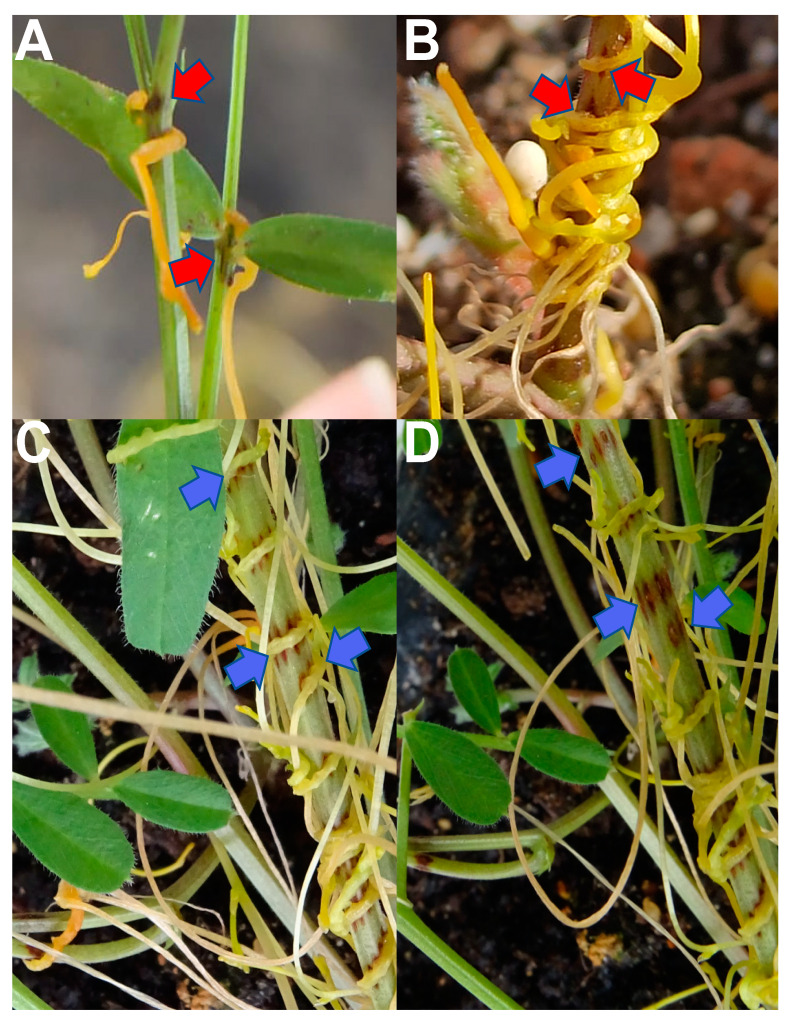
Hypersensitive-like response developed on *Vicia sativa* accession Vs.1 in response to *Cuscuta campestris* infection. Red arrows point at hypersensitive-like response at *Vicia-Cuscuta* interface in (**A**) *V. sativa* lateral branch and (**B**) *V. sativa* base of the main stem. Blue arrows point at hypersensitive-like response at *Vicia-Cuscuta* interface (**C**) before and (**D**) after *Cuscuta* haustorium was manually removed for a better visualization of the resistance response.

In contrast to the results observed in the screening of *V. ervilia* accessions, the screening of 135 accessions of *V. sativa* revealed that resistance is very scarce, but exists in this legume species (Table 2). Each *V. sativa* accession was planted and inoculated as described for *V. ervilia* (see Materials and Methods section). As occurred in *V. ervilia* plants, *C. campestris* seedlings were able to coil quickly around the stem of all *V. sativa* accessions, indicating the absence of a mechanism of repellency in *V. sativa* plants against *C. campestris*. Table 2 also shows data on the posthaustorial growth of nine day-old *C. campestris* recorded in the 23 most resistant *V. sativa* accessions in which at least 50% of the plants had resisted the penetration of the haustorium and inhibited the posthaustorial growth. Accession Vs.1 and Vs.4 were able to resist in all their plants tested the infection of nine day-old *C. campestris*. The remaining 112 *V. sativa* accessions allowed in more than 50% of their plants the development of posthaustorial growth in nine day-old *C. campestris* seedlings. Fourteen days later, *C. campestris* seedlings were able to penetrate all the accessions including those accessions that resisted the infection, however, contrary to what happened in the *V. ervilia* collection, a darkening at the penetration site resembling a hypersensitive-like response (Figure 2A–D) was observed 14 days later in these *V. sativa* accessions with this darkening visible in 100% of the plants in accessions Vs.1, Vs.4, Vs.6, Vs.9, Vs.68, and Vs.84.

### 2.3. Confirmation of Resistant Vicia sativa Phenotypes

Table 3 shows the results of a second greenhouse screening performed on accessions Vs.1, Vs.4, Vs.6, Vs.9, Vs.68, and Vs.84 to confirm the resistant response identified by the first greenhouse screening. In addition, we included the accessions Vs.11, Vs.51, and Vs.80 that also showed hypersensitive-like response but it was observed that plant segregation being the resistant phenotype was only visible in some of their plants, possibly due to lack of homogeneity of these *V. sativa* accessions stored in the germplasm collections of the Germplasm banks. Two susceptible accessions, Vs.8 and Vs.121, without induction of the hypersensitive-like response were also included. In this screening, we confirmed the delay of *C. campestris* early development and the induction of hypersensitive-like response in later stages of *C. campestris* in all plants of accessions Vs.1, Vs.4, and Vs.6 while these resistant responses were not observed in the susceptible accession Vs.8. The susceptible accession Vs.121 was very sensitive to *Cuscuta* infection and died before the evaluation date. Accessions Vs.9, Vs.11, Vs.51, Vs.68, Vs.80, and Vs.84 showed segregation being the resistant response only present in some of their plants, indicating that these accessions were not genetically homogeneous for this resistant character. Resistant plants were selected and multiplied in the absence of pollinators to initiate a breeding program for resistance against *Cuscuta* infection.

### 2.4. Effects of Resistant Response in Vicia sativa and Cuscuta campestris Trophic Relations

**Figure 3:**
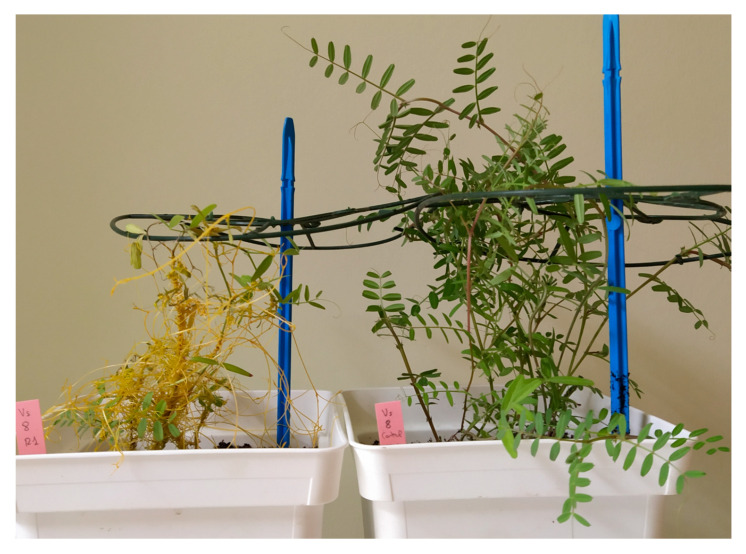
Effects of *Cuscuta campestris* infection on biomass of susceptible *Vicia sativa* accession Vs.8. (**left**) in comparison with uninfected Vs.8. plants (**right**).

A third experiment was carried out in *V. sativa* to characterize the effect that the resistance response has in the trophic relations during infection between *V. sativa* accessions and *Cuscuta* (Table 4). The weight of dry biomass of host root, host aboveground tissues, and *Cuscuta* tissues were estimated in resistant accessions Vs.1, Vs.4, Vs.68, and Vs. 84 and compared with the dry weight of corresponding compartments in susceptible accessions Vs.8 and Vs.121. In addition, root and aboveground biomass of each accession was determined in uninfected control plants. The extreme susceptibility of accession Vs.121 to *Cuscuta* infection caused the death of the infected Vs.121 plants before the harvesting date. *Cuscuta* infection greatly reduced the total biomass of susceptible plants. The total dry weight of *V. sativa* accessions Vs.8 and Vs.121 plants infected with *Cuscuta* was respectively reduced by 84.3% and 79.3% in comparison with their corresponding uninfected control plants. The biomass reduction of infected plants was visible both in aerial biomass (86.4% and 82.0%) and root biomass (81.3% and 75.3%), respectively, for the Vs.8 and Vs.121 accessions (Figure 3). The ratio of parasite/host shoot dry biomass was 2.89 and 2.22, respectively, for Vs.8 and Vs.121 accessions. The biomass gain of *Cuscuta* did not account for the difference in biomass between infected and uninfected *V. sativa* plants. Combining *V. sativa* and *Cuscuta* total biomass revealed that the combined biomass of the infected system in accessions Vs.8 and Vs.121 was respectively 56.9% and 55.5% lower than that of the Vs.8 and Vs.121 uninfected plants. Besides the reduction in host biomass, *Cuscuta* also modified host allometric relationships among above and belowground organs. In uninfected *V. sativa* susceptible accessions Vs.8 and Vs.121, the percentage of dry weight allocated in aboveground organs with respect to total *V. sativa* dry weight of the entire plant was 61.0% and 62.1%, respectively. *Cuscuta* infection reduced this percentage to 20.7% in accession Vs.8 and to 24.3% in accession Vs.121. When adding the parasite sink as an aerial organ of the infected system, the ratio of combined host and parasite aboveground dry biomass to total combined biomass increased up to 79.1% for Vs.8 and 77.2% for Vs.121 infected plants. 

The *Cuscuta* biomass gain and the effect of *Cuscuta* infection on the reduction of *V. sativa* aboveground and root dry weight, biomass loss of infected system, and changes in allometric relationships were not significantly different between susceptible accessions Vs.8 and Vs.121 and the accessions responding with hypersensitive-like reactions Vs.4, Vs.68, and Vs.84. In contrast, the resistant response observed in accession Vs.1 was effective in limiting the growth of *C. campestris,* being the ratio of parasite/host shoot dry biomass, to only 0.59, significantly lower than the ratio of parasite/host shoot dry biomass in the rest of the *V. sativa* accessions. The reduction of parasite growth by the resistant mechanism in Vs.1 had positive effects on the host biomass, and the reduction of Vs.1 biomass by infection was significantly lower than the biomass reduction observed in the rest of the *V. sativa* accessions. Limited biomass loss due to infection in Vs.1 was observed both in the host aboveground and root biomass and in the combined biomass of the infected system in comparison with the susceptible accessions (Table 4). 

## 3. Discussion

From the screening of 135 accessions of *V. sativa* and 154 accessions of *V. ervilia*, we have identified in this work a resistant phenotype against *C. campestris* infection in *V. sativa* accessions. The resistant phenotype was observed after *Cuscuta* germination, coiling and prehaustorium development on the host, with a darkening of the host–parasite contact site observed as a hypersensitive-like response. Hypersensitive-like response at the *Vicia*-parasite contact has been previously observed against the root parasitic weed [30,31], but never against *Cuscuta* infection. The observed phenotype was exhibited by all plants in Vs.1, Vs.4, and Vs.6 accessions, while a degree in plant segregation in their hypersensitive-like response was observed in Vs.9, Vs.11, Vs.51, Vs.68, Vs.80, and Vs.84, being visible only in some of their plants possibly due to lack of homogeneity of these *V. sativa* accessions stored in the germplasm collections of the Germplasm banks. No resistant accessions were found in *V. ervilia* despite the large number of genotypes from diverse worldwide origins in the collection used for the screening. Resistance to *C. campestris* infection is very rare in cultivated species and to date, no varietal differences have been described in the responses to *C. campestris* infection in the majority of susceptible crops with few exceptions like in the greenhouse study of a collection of chickpea genotypes, which identified two resistant genotypes ‘ICCV95333’ and ‘Hazera4’ [22]. The resistant mechanism identified in chickpea by Goldwasser et al. [22] was different from that identified in our work in *Vicia sativa* genotypes and described as a seedling repellency-based mechanism after prehaustoria differentiation.

Both susceptible and resistant *V. sativa* plants infected with *Cuscuta* accumulated less dry matter than uninfected control plants, however, the loss of host biomass was significantly lower in resistant genotypes. The biomass accumulated by the parasite and the ratio of parasite/host aboveground dry biomass was much lower in resistant than in susceptible *V. sativa* plants. These observations agree with those reported in *C. campestris*–chickpea interactions [22], but they contrast to those reported in *C. reflexa*–host interactions [34,35]. The difference in host biomass between infected and uninfected plants did not equal that accumulated by the parasite, which agreed with *Striga–*host interactions [36,37], but disagreed with *Orobanche*–host interactions [38,39]. Besides the reduction in host biomass, *Cuscuta* also induced changes in the partitioning of dry weight between aboveground and belowground organs within the host, reducing the percentage of total *V. sativa* biomass allocated in aboveground organs with respect to total *V. sativa* dry weight. These findings are in contrast to those observed in *Striga*, which do not tend to cause large changes in the proportion of dry matter partitioned between photosynthetic and non-photosynthetic organs [40,41] and to those observed in *Orobanche*, which increases the ratio of photosynthetic to non-photosynthetic organs [38]. Resistance to *Cuscuta* parasitism in accession Vs.1 was absolute, *Cuscuta* development was strongly inhibited, and host biomass reduction was strongly inhibited. 

## 4. Materials and Methods

### 4.1. Plant Material

*Cuscuta* seeds were collected in June 2018 from mature *Cuscuta campestris* plants parasitizing chickpea in a field at the IFAPA Center Alameda del Obispo of Córdoba, Spain. *Cuscuta* seeds were separated from dry capsules using a combination of winnowing with a fan and sifting with a 0.6 mm mesh-size sieve (Filtra, Barcelona, Spain). *Cuscuta* seeds were stored dry in the dark at room temperature until use for this work in the spring of 2020. 

Screening for *Cuscuta* resistant genotypes was performed in germplasm collections of two legume species, bitter vetch (*Vicia ervilia* (L.) Willd.) and common vetch (*Vicia sativa* L*. ssp. sativa*). The collection of bitter vetch was formed by 154 accessions kindly provided by the International Center for Agricultural Research in the Dry Areas (ICARDA) by the U.S. National Plant Germplasm System (USDA). The collection of common vetch was formed by 136 accessions kindly provided by Centro de Recursos Fitogenéticos (Madrid, Spain), the Genetic Resources Unit of ICARDA (International Center for Agricultural Research in the Dry Areas, Aleppo, Syria), USDA-ARS, and the Genetic Resources Unit of Hellenic Agricultural Organization, Industrial & Fodder Crops Inst. (Thessaloniki, Greece).

### 4.2. Greenhouse Screening of Vicia ervilia and Vicia sativa Germplasm Collection 

In a greenhouse at the IFAPA Research Center (Centro Alameda del Obispo, Córdoba Spain), 867 pots of 10.3 cm each side and 13.2 cm high containing 1 L of 1/1 sand and peat proportion were prepared for the screening of 154 accessions of bitter vetch and 135 accessions of common vetch. Three seeds of each accession of each vetch species were sown in triplicate pots in a complete randomized design. Vetch plants were grown for 16 days (10 °C–27 °C min and max temperature) before *Cuscuta* inoculation. They were irrigated with tap water every two days. 

To promote *Cuscuta* germination, the hard coat of *Cuscuta* seeds was eliminated by scarification with sulfuric acid during 45 min, followed by throughout rinsing with sterile distilled water and air-dried. Scarified *Cuscuta* seeds were spread in wet filter paper inside Petri dishes and allowed to germinate in the dark during two days at 23 °C. Then, four germinated *Cuscuta* seedlings were manually placed using tweezers on the soil surface surrounding each of the three vetch plants per pot at 2 cm distance of the vetch stem. Seven days later, the *Cuscuta* seedlings were visually inspected and classified as either (i) unattached *Cuscuta* seedling, (ii) attached *Cuscuta* seedling without adhesion disks, (iii) attached *Cuscuta* seedling with adhesion disks without posthaustorial growth, and (iv) posthaustorial growth from the adhesion disks. At the end of the experiment, mature haustoria were inspected for hypersensitive-like response at the vetch–*Cuscuta* interface. 

### 4.3. Confirmation of Resistant Phenotypes

A resistant phenotype was identified in some *V. sativa* accessions during the first screening. The resistant phenotype was characterized as a delay in the development of posthaustorial growth in nine day-old *C. campestris* seedlings (Figure 1) and a subsequent darkening resembling a hypersensitive-like response at the penetration site of 23 day-old *C. campestris* (Figure 2). A second greenhouse screening was performed to confirm the resistant phenotypes in common vetch accessions, Vs.1, Vs.4, Vs.6, Vs.7, Vs.9, Vs.11, Vs.19, Vs.51, Vs.68, Vs.80, and Vs.84, identified by the first greenhouse screening. In addition, two highly susceptible common vetch accessions, Vs.8 and Vs.121, were included as susceptible controls. The experimental design, common vetch and *Cuscuta* cultivation, and resistance phenotyping were performed as described above.

### 4.4. Effects of Cuscuta Infection in Resistant and Susceptible Common Vetch Accessions

A third experiment was carried out to characterize the effect of *Cuscuta* infection on the biomass of common vetch resistant and susceptible accessions. In the greenhouse, 36 pots of 18 cm each side and 25.5 cm high containing 5.5 L of 1/1 sand and peat proportion were prepared to sow four resistant accessions (Vs.1, Vs.4, Vs. 68, and Vs.84) and two susceptible controls (Vs.8 and Vs.121). *Cuscuta* seeds were scarified, germinated, and manually inoculated on three pots per vetch accession as described before. Each accession was cultivated in three pots without *Cuscuta* as uninfected controls. At the end of the cultivation cycle, the consequences in common vetch productivity of *Cuscuta* parasitism was estimated by recording separately in each pot the *Cuscuta* and host biomass [39]. For each common vetch accession, *Cuscuta* dry weight, host aboveground, and host root tissue were collected separately and carried to the laboratory. Samples were dried at 70 °C for 48 hours and each biomass compartment weighed independently to determine five parameters: (i) host aboveground dry weight; (ii) host root dry weight; (iii) *Cuscuta* dry weight; (iv) total host biomass (aboveground and root host dry weight); and (v) combined biomass (total host and *Cuscuta* dry weight). Using these parameters, varietal differences in the severity of *Cuscuta* infection were determined with (i) *Cuscuta* dry weight, and the (ii) ratio of parasite/host shoot dry weight. Varietal differences in the effect of *Cuscuta* infection in allometric relationships were determined by calculating the (i) host aboveground biomass index (ratio of aboveground host dry matter/total host dry matter), and (ii) the combined aboveground biomass index (ratio of aboveground host and *Cuscuta* dry matter/combined dry matter). Varietal differences in the *Cuscuta*-induced changes in productivity were studied analyzing four parameters: (i) reduction in total host biomass (ratio of total host biomass of infected plants/total host biomass of uninfected plants); (ii) reduction in aboveground host biomass (ratio of aboveground host dry matter of infected plants/aboveground host dry matter of uninfected plants); (iii) reduction in host root biomass (ratio of host root dry matter of infected plants/host root dry matter of uninfected plants); and (iv) reduction in combined biomass (ratio of total combined host–parasite biomass in infected plants/total host biomass of uninfected plants).

### 4.5. Statistical Analysis

The experimental design was randomized complete blocks. Percentage data were transformed with arcsin (√(x/100) before analysis. Analysis of variance (one-way ANOVA) was applied to replicate data, with accession as the main factor using Statistix 9.1 software (Analytical software, Tallahassee, FL, USA). The significance of mean differences between each genotype against the control was evaluated by the two-sided Dunnett test. The significance of mean differences among genotypes was evaluated by the least significant difference (LSD) (*p* < 0.05).

## 5. Conclusions

The majority of *V. sativa* and *V. ervilia* accessions studied were susceptible to *Cuscuta* infection. We identified the *V. sativa* accession Vs.1 with high resistance to infection. The resistant phenotype is characterized by a hypersensitive-like response, resulting in inhibition of *Cuscuta* growth and reduction of biomass loss of infected Vs.1 plants. To the best of our knowledge, this is the first identification of *Cuscuta* resistance in *V. sativa* genotypes. Further histological and biochemical studies will continue this research to elucidate the exact mechanism involved. 

## Figures and Tables

**Table 1:** Compatibility of *Cuscuta campestris* seedlings with a collection of 154 accessions of *Vicia ervilia*. Data expressed as the (†) percentage of *V. ervilia* plants with *Cuscuta* seedlings coiled around their stems; (††) percentage of *V. ervilia* plants with *Cuscuta* haustorium formed and posthaustorial growth emerging from the penetration site; (†††) percentage of *V. ervilia* plants showing hypersensitive-like response at the penetration site. Analysis of variance was applied to replicate data. Differences among genotypes with the susceptible genotype Ve.1 were assessed by Dunnett’s test. **, and *** indicate significant differences at *p* < 0.05, 0.01, and 0.001, respectively.

Accession Experimental Code	Accession Original Code	Accession Origin	9 Days-Old *Cuscuta*		20 Days-Old *Cuscuta*	40 Days-Old *Cuscuta*
			Coiling (%) (†)	Haustorium Formation with Posthaustorial Growth (%) (††)	Haustorium Formation with Posthaustorial Growth (%) (††)	Hypersensitive-Like Response (%) (†††)
Ve.1	IFVE 2799	Unknown	100	100.0	100	0.0
Ve.7	IFVE 2852	Unknown	100	33.3 ***	100	0.0
Ve.63	PI220207	Afghanistan	100	38.9 ***	100	0.0
Ve.70	PI222215	Afghanistan	100	41.7 ***	100	0.0
Ve.71	PI222754	Iran	100	30.6 ***	100	0.0
Ve.73	PI223299	Afghanistan	100	41.7 ***	100	0.0
Ve.78	PI223547	Afghanistan	100	45.8 **	100	0.0
Ve.79	PI227052	Iran	100	38.9 ***	100	0.0
Ve.81	PI227878	Iran	100	45.8 **	100	0.0
Ve.89	PI239916	Iran	100	25.0 ***	100	0.0
Ve.90	PI239917	Iran	100	33.3 ***	100	0.0
Ve.91	PI251199	Yugoslavia	100	19.4 ***	100	0.0
Ve.95	PI253999	Afghanistan	100	41.7 ***	100	0.0
Ve.113	PI381064	Iran	100	44.4 **	100	0.0
Ve.115	PI388900	Turkey	100	41.7 ***	100	0.0
Ve.116	PI393846	Canada	100	31.9 ***	100	0.0
Ve.118	PI393849	Canada	100	38.9 ***	100	0.0
Ve.119	PI393850	Canada	100	48.6 **	100	0.0
Ve.124	PI420950	Jordan	100	30.6 ***	100	0.0
Ve.130	PI515981	Turkey	100	27.8 ***	100	0.0
Ve.136	PI518458	Spain	100	0.0 ***	100	0.0
Ve.145	PI518468	Spain	100	41.7 ***	100	0.0
Ve.151	PI628280	Turkey	100	44.4 **	100	0.0
Ve.159	PI628297	Bulgaria	100	37.5 **	100	0.0
Ve.163	PI628320	Iran	100	45.8 **	100	0.0
Remaining 129 accessions			100	79.1	100	0.0

**Table 2:** Compatibility of *Cuscuta campestris* seedlings with a collection of 135 accessions of *Vicia sativa*. Data expressed as the (†) percentage of *V. sativa* plants with *Cuscuta* seedlings coiled around their stems; (††) percentage of *V. sativa* plants with *Cuscuta* haustorium formed and posthaustorial growth emerging from the penetration site; (†††) percentage of *V. sativa* plants showing hypersensitive-like response at the penetration site. Analysis of variance was applied to replicate data. Differences among genotypes with the susceptible genotype Vs.8 were assessed by Dunnett’s test. *, **, and *** indicate significant differences at *p* < 0.05, 0.01, and 0.001, respectively; ^ns^ indicates no significant difference when comparing each common vetch accession with the Vs.8.

Accession Experimental Code	Accession Original Code	Accession Origin	9 Days-Old *Cuscuta*		20 Days-Old *Cuscuta*	40 Days-Old *Cuscuta*
			Coiling (%) (†)	Haustorium Formation with Posthaustorial Growth (%) (††)	Haustorium Formation with Posthaustorial Growth (%) (††)	Hypersensitive-Like Response (%) (†††)
Vs. 8	IFVS 1852	Turkey	100.0	88.9	100.0	0.0
Vs.1	IFVS 505	Afghanistan	100.0	0.0 ***	100.0	100.0 ***
Vs.2	IFVS 1625	Turkey	100.0	22.2 ***	100.0	0.0 ^ns^
Vs.3	IFVS 1626		100.0	100.0 ^ns^	100.0	0.0 ^ns^
Vs.4	IFVS 1643	Turkey	100.0	0.0 ***	100.0	100.0 ***
Vs.6	IFVS 1710	Turkey	100.0	22.2 ***	100.0	100.0 ***
Vs.7	IFVS 1803	Turkey	100.0	88.9 ^ns^	100.0	33.3 ***
Vs.9	IFVS 2006	Turkey	100.0	44.4 **	100.0	100.0 ***
Vs.11	IFVS 2911	Turkey	100.0	50.0 *	100.0	50.0 ***
Vs. 16	MEZQUITA	Spain (Córdoba)	100.0	100.0 ^ns^	100.0	0.0 ^ns^
Vs.21	PI284080		100.0	88.9 ^ns^	100.0	0.0 ^ns^
Vs. 24	PI284402		100.0	100.0 ^ns^	100.0	0.0 ^ns^
Vs.38	BGE001155	Spain (Segovia)	100.0	100.0 ^ns^	100.0	0.0 ^ns^
Vs.50	BGE002028	Spain (Palencia)	100.0	100.0 ^ns^	100.0	0.0 ^ns^
Vs.51	BGE003718	Spain (Zamora)	100.0	100.0 ^ns^	100.0	44.4 ***
Vs.57	BGE004313	Spain (Málaga)	100.0	77.8 ^ns^	100.0	0.0 ^ns^
Vs.58	BGE004314	Spain (Málaga)	100.0	100.0 ^ns^	100.0	0.0 ^ns^
Vs.60	BGE004340	Spain (Málaga)	100.0	100.0 ^ns^	100.0	55.6 ***
Vs.68	BGE004360	Spain (Málaga)	100.0	50.0 **	100.0	100.0 ***
Vs.80	BGE004388	Spain (Málaga)	100.0	88.9 ^ns^	100.0	33.3 ***
Vs.84	BGE004394	Spain (Málaga)	100.0	44.4 **	100.0	100.0 ***
Vs.109	BGE014916	Spain (Granada)	100.0	88.9 ^ns^	100.0	0.0 ^ns^
Vs. 121	BGE016971	Spain (Toledo)	100.0	100.0 ^ns^	100.0	0.0 ^ns^
Remaining 112 accessions			100.0	100.0	100.0	0.0

**Table 3:** Compatibility of *Cuscuta campestris* seedlings with a selected collection of resistant and susceptible *Vicia sativa* accessions. Data expressed as the (†) percentage of *V. sativa* plants with *Cuscuta* seedlings coiled around their stems; (††) percentage of *V. sativa* plants with *Cuscuta* adhesive disks and posthaustorial growth emerging from the penetration site; (†††) percentage of *Vicia sativa* plants showing hypersensitive-like response at the penetration site. Analysis of variance was applied to replicate data. Differences among genotypes with the susceptible genotype Vs.8 were assessed by Dunnett’s test. *, **, and *** indicate significant differences at *p* < 0.05, 0.01, and 0.001, respectively; ^ns^ indicates no significant difference when comparing each common vetch accession with the Vs.8.

Accession Experimental Code	Accession Original Code	Accession Origin	9 Days-Old *Cuscuta*			20 Days-Old *Cuscuta*		40 Days-Old *Cuscuta*
			Coiling (%) (†)	Haustorium Formation with Posthaustorial Growth (%) (††)	Hypersensitive-Like Response (%) (†††)	Haustorium Formation with Posthaustorial Growth (%) (††)	Hypersensitive-Like Response (%) (†††)	Hypersensitive-Like Response (%) (†††)
Vs.8	IFVS 1852	Turkey	100	88.9	0.0	100	0.0	0.0
Vs.1	IFVS 505	Afghanistan	100	0.0 ***	0.0	100	100.0	100.0 ***
Vs.4	IFVS 1643	Turkey	100	33.3 ***	0.0	100	0.0	100.0 ***
Vs.6	IFVS 1710	Turkey	100	22.2 ***	0.0	100	0.0	100.0 ***
Vs.9	IFVS 2006	Turkey	100	100.0 ^ns^	0.0	100	0.0	77.8 ***
Vs.11	IFVS 2911	Turkey	100	22.2 ***	0.0	100	0.0	22.2 ^ns^
Vs.51	BGE003718	Spain (Zamora)	100	100.0 ^ns^	0.0	100	0.0	71.4 ***
Vs.68	BGE004360	Spain (Málaga)	100	53.9 *	0.0	100	0.0	44.4 **
Vs.80	BGE004388	Spain (Málaga)	100	33.3 ***	0.0	100	0.0	50.0 ***
Vs.84	BGE004394	Spain (Málaga)	100	66.7 ^ns^	0.0	100	0.0	66.7 ***
Vs. 121	BGE016971	Spain (Toledo)	100	100.0 ^ns^	0.0	100	0.0	0.0 ^ns^

**Table 4:** Varietal differences in the severity of *Cuscuta* infection were determined with (i) *Cuscuta* dry matter, and the (ii) ratio of parasite/host shoot dry biomass. Varietal differences in the effect of *Cuscuta* infection in allometric relationships were determined by calculating the (i) host aboveground biomass index (ratio of aboveground host dry matter/total host dry matter), and (ii) the combined aboveground biomass index (ratio of aboveground host and *Cuscuta* dry matter/total combined host–parasite dry matter). Varietal differences in the *Cuscuta*-induced changes in productivity were studied analyzing four parameters: (i) reduction in total host biomass (ratio of total host biomass of infected plants/total host biomass of uninfected plants); (ii) reduction in aboveground host biomass (ratio of aboveground host dry matter of infected plants/aboveground host dry matter of uninfected plants); (iii) reduction in host root biomass (ratio of host root dry matter of infected plants/host root dry matter of uninfected plants); (iv) reduction in combined biomass (ratio of total combined host–parasite dry matter in infected plants/total host biomass of uninfected plants). Analysis of variance was applied to replicate data. Differences among genotypes with the susceptible genotype Vs.8 were assessed by Dunnett’s test. *, **, and *** indicate significant differences at *p* < 0.05, 0.01, and 0.001, respectively; ^ns^ indicates no significant difference when comparing each common vetch accession with the Vs.8. Least significant difference (LSD) value (*p*< 0.05) is provided for comparison among accessions.

Accession Experimental Code	Accession Original Code	Accession Origin	*Cuscuta* Infection Severity		Allometric Relationships			Reduction of Dry Weight in Infected Plant Relative to Uninfected Plants (%)			
			*Cuscuta* Dry Matter (g)	Relative *Cuscuta* Biomass	Host Aboveground Biomass Index (%)		Combined Aboveground Biomass Index (%)	Reduction in Total Host Biomass (%)	Reduction in Host Aboveground Biomass (%)	Reduction in Host Root Biomass (%)	Reduction in Combined Biomass (%)
					Uninfected	Infected					
Vs.8	IFVS 1852	Turkey	0.39	2.89	61.0	20.7	79.1	84.3	86.4	81.3	60.4
Vs.1	IFVS 505	Afghanistan	0.19 *	0.51 ***	66.4 ^ns^	48.0 ***	71.4 ^ns^	47.6 ***	49.4 ***	44.3 ***	31.1 *
Vs.4	IFVS 1643	Turkey	0.50 ^ns^	1.79 *	49.7 ^ns^	28.2 ^ns^	78.5 ^ns^	68.2 *	63.6 **	72.8 ^ns^	36.3 ^ns^
Vs.68	BGE004360	Spain (Málaga)	0.38 ^ns^	2.30 ^ns^	56.6 ^ns^	23.6 ^ns^	77.0 ^ns^	79.8 ^ns^	81.9 ^ns^	77.0 ^ns^	57.4 ^ns^
Vs.84	BGE004394	Spain (Málaga)	0.41 ^ns^	2.04 ^ns^	59.8 ^ns^	25.6 ^ns^	77.2 ^ns^	81.1 ^ns^	83.2 ^ns^	77.9 ^ns^	60.6 ^ns^
Vs.121	BGE016971	Spain (Toledo)	0.24 ^ns^	2.22 ^ns^	62.1 ^ns^	24.3 ^ns^	77.2 ^ns^	79.3 ^ns^	82.0 ^ns^	75.3 ^ns^	55.5 ^ns^
LSD (0.05)			0.19	0.79	16.34	6.87	6.02	8.38	9.09	10.03	19.06

## Data Availability

The data presented in this study are available on request from the corresponding author.

## References

[1] Huang Y.F., Gao XL., Nan Z.B., Zhang Z.X. (2017). Potential value of the common vetch (*Vicia sativa* L.) as an animal feedstuff: A review. J. Anim. Physiol. Anim. Nutr..

[2] Mao Z.X., Fu H., Nan Z.B., Wan C.G. (2015). Fatty acid, amino acid, and mineral composition of four common vetch seeds on Qinghai-Tibetan plateau. Food Chem..

[3] Dhima K.V., Lithourgidis A.S., Vasilakoglou I.B., Dordas C.A. (2007). Competition indices of common vetch and cereal intercrops in two seeding ratio. Field Crop. Res..

[4] Francis C.M., Enneking D., Abd El Moneim A.M. When and where will vetches have an impact as seed legumes?. Proceedings of the 3rd International Food Legume Research Conference.

[5] FAOSTAT (2020). Food and Agriculture Organization of United Nations. Rome. http://faostat3.fao.org.org.

[6] MARM (2019). Anuario de Estadística Agroalimentaria.

[7] Anil L., Park J., Phipps R.H., Miller F.A. (1998). Temperate intercropping of cereals for forage: A review of the potential for growth and utilization with particular reference to the UK. Grass Forage Sci..

[8] Atis I., Kokten K., Hatipoglu R., Yilmaz S., Atak M., Can E. (2012). Plant density and mixture ratio effects on the competition between common vetch and wheat. Aust. J. Crop Sci..

[9] Ladizinsky G. (1998). Plant Evolution under Domestication.

[10] Zohary D., Hopf M. (2000). Domestication of Plants in the Old World.

[11] Vasilakoglou I., Dhima K., Lithourgidis A., Eleftherohorinos I. (2008). Competitive ability of winter cereal-common vetch intercrops against sterile oat. Exp. Agric..

[12] Ministry of Agriculture and Fisheries and Food Agri-Food Statistics Yearbook 2019. Crop Areas and Yields. Grain Legumes. Bitter Vetch. https://www.mapa.gob.es/es/estadistica/temas/publicaciones/anuario-de-estadistica/2019/default.aspx?parte=3&capitulo=07&grupo=2&seccion=8.

[13] Enneking D., Lahlou A., Noutfia A., Bounejmate M. (1995). A note on *Vicia ervilia* cultivation utilisation and toxicity in Morocco. Al Awamia.

[14] Dong R., Dong D., Luo D., Zhou Q., Chai X., Zhang J., Xie W., Liu W., Dong Y., Wang Y. (2017). Transcriptome analyses reveal candidate pod shattering-associated genes involved in the pod ventral sutures of common vetch (*VICIA sativa* L.). Front. Plant Sci..

[15] Pérez-de-Luque A., Lozano M.D., Cubero J.I., González-Melendi P., Risueño M.C., Rubiales D. (2006). Mucilage production during the incompatible interaction between *Orobanche crenata* and *Vicia sativa*. J. Exp. Bot..

[16] Fernández-Aparicio M., Sillero J.C., Rubiales D. (2009). Resistance to broomrape species (*Orobanche* spp.) in common vetch (*Vicia sativa* L.). Crop Prot..

[17] González-Verdejo C.I., Fernández-Aparicio M., Córdoba E.M., López-Ráez J.A., Nadal S. (2020). Identification of *Vicia ervilia* germplasm resistant to *Orobanche crenata*. Plants (Basel).

[18] González-Verdejo C.I., Fernández-Aparicio M., Córdoba E.M., Nadal S. (2021). Resistance against *Orobanche crenata* in Bitter Vetch (*Vicia ervilia*) Germplasm Based on Reduced Induction of Orobanche Germination. Plants (Basel).

[19] Karadavut U., Bakoglu A., Tutar H., Kokten K., Yilmaz H.S. (2017). Prediction of dry matter accumulation in bitter vetch. Legume Res..

[20] Kuijt J. (1969). The Biology of Parasitic Flowering Plants.

[21] Dawson J.H., Musselman L.J., Wolswinkel P., Dörr I. (1994). Biology and control of Cuscuta. Rev. Weed Sci..

[22] Goldwasser Y., Miryamchik H., Sibony M., Rubin B. (2012). Detection of resistant chickpea (*Cicer arietinum*) genotypes to *Cuscuta campestris* (field dodder). Weed Res..

[23] Christensen N.M., Dorr I., Hansen M., van der Kooij T.A.W., Schulz A. (2003). Development of *Cuscuta* species on a partially incompatible host: Induction of xylem transfer cells. Protoplasma.

[24] Cudney D.W., Orloff S.B., Reints J.S. (1992). An integrated weed management for the control of dodder (*Cuscuta indecora*) in alfalfa (*Medicago sativa*). Weed Technol..

[25] Parker C., Riches C.R. (1993). Parasitic Weeds of the World: Biology and Control.

[26] Fernández-Aparicio M., Delavault P., Timko M. (2020). Management of infection by parasitic weeds: A 434 review. Plants.

[27] Guza C.J. (2000). Weed Control with Glyphosate and Glufosinate in Herbicide-Resistant Sugarbeets (*Beta vulgaris* L.). Master’s Thesis.

[28] Nadler-Hassar T., Rubin B. (2003). Natural tolerance of *Cuscuta campestris* to herbicides inhibiting amino acid biosynthesis. Weed Res..

[29] Nadler-Hassar T., Shaner D.L., Nissen S., Westra P., Rubin B. (2009). Are herbicide-resistant crops the answer to controlling *Cuscuta*?. Pest Manag. Sci..

[30] Goldwasser Y., Hershenhorn J., Plakhine D., Kleifeld Y., Rubin B. (1999). Biochemical factors involved in vetch resistance to Orobanche aegyptiaca. Physiol. Mol. Plant Pathol..

[31] Goldwasser Y., Plakhine D., Kleifeld Y., Zamski E., Rubin B. (2000). The differential susceptibility of vetch (*Vicia* spp.) to *Orobanche aegyptiaca*: Anatomical studies. Ann. Bot..

[32] Pérez-de-Luque A., Rubiales D., Cubero J.I., Press M.C., Scholes J., Yoneyama K., Takeuchi Y., Plakhine D., Joel D.M. (2005). Interaction between Orobanche crenata and its host legumes: Unsuccessful haustorial penetration and necrosis of the developing parasite. Ann. Bot..

[33] Gil J., Martín L.M., Cubero J.I. (1987). Genetics of resistance in Vicia sativa L. to Orobanche crenata Forsk. Plant Breed..

[34] Jeschke W.D., Hilpert A. (1997). Sink-stimulated photosynthesis and sink-dependent increase in nitrate uptake: Nitrogen and carbon relations of the parasitic association *Cuscuta reflexa*–*Ricinus communis*. Plant Cell Environ..

[35] Jeschke W.D., Baig A., Hilpert A. (1997). Sink-stimulated photosynthesis, increased transpiration and increased demand-dependent stimulation of nitrate uptake: Nitrogen and carbon relations in the parasitic association *Cuscuta reflexa*–*Coleus blumei*. J. Exp. Bot..

[36] Graves J.D., Press M.C., Smith S., Stewart G.R. (1992). The carbon economy of the association between cowpea and the parasitic angiosperm *Striga gesnerioides*. Plant Cell Environ..

[37] Gurney A.L., Press M.C., Ransom J.L. (1995). The parasitic angiosperm *Striga hermonthica* can reduce photosynthesis of its sorghum and maize hosts in the field. J. Exp. Bot..

[38] Hibberd J.M., Quick W.P., Press M.C., Scholes J.D. (1998). Can source sink relation sex plain responses of tobacco to infection by the root holoparasitic angiosperm *Orobanche cernua*?. Plant Cell Environ..

[39] Fernández-Aparicio M., Flores F., Rubiales D. (2016). The effect of *Orobanche crenata* infection severity in faba bean, field pea and grass pea productivity. Front. Plant Sci..

[40] Press M.C., Stewart G.R. (1987). Growth and photosynthesis in Sorghum bicolor infected with *Striga hermonthica*. Ann. Bot..

[41] Cechin I., Press M.C. (1993). Nitrogen relations of the *Sorghum*-*Striga hermonthica* host-parasite association: Growth and photosynthesis. Plant Cell Environ..

